# High-Power Laser Therapy Improves Healing of the Equine Suspensory Branch in a Standardized Lesion Model

**DOI:** 10.3389/fvets.2020.00600

**Published:** 2020-09-03

**Authors:** Mathilde Pluim, Ann Martens, Katrien Vanderperren, René van Weeren, Maarten Oosterlinck, Jeroen Dewulf, Mimoun Kichouh, Bert Van Thielen, Marc H. W. Koene, Antonio Luciani, Lukas Plancke, Cathérine Delesalle

**Affiliations:** ^1^Department of Virology, Parasitology and Immunology, Research Group of Comparative Physiology, Faculty of Veterinary Medicine, Ghent University, Ghent, Belgium; ^2^Tierklinik Lüsche GmbH, Bakum, Germany; ^3^Department of Surgery and Anaesthesiology of Domestic Animals, Faculty of Veterinary Medicine, Ghent University, Ghent, Belgium; ^4^Department of Veterinary Medical Imaging and Small Animal Orthopedics, Faculty of Veterinary Medicine, Ghent University, Ghent, Belgium; ^5^Department of Clinical Sciences, Utrecht University, Utrecht, Netherlands; ^6^Unit of Veterinary Epidemiology, Department of Obstetrics, Reproduction and Herd Health, Faculty of Veterinary Medicine, Ghent University, Ghent, Belgium; ^7^Radiology Department, Brussels University Hospital, Brussels, Belgium; ^8^Odisee Hogeschool, Campus Terranova, Training Center for Imaging Technologists, Brussels, Belgium

**Keywords:** laser, suspensory, ligament, equine, tendon, MRI, ultrasound, high-power

## Abstract

High-power laser therapy is often used as a treatment for human sport injuries but controlled standardized studies on its efficacy are lacking. The technique has also been introduced in the equine field and recently promising results were reported in a retrospective study focusing on 150 sporthorses suffering from tendinopathy and desmopathy of the SDFT, DDFT, suspensory ligament, and suspensory branches. The goal of the present study was to evaluate the effect of high-power laser in a standardized lesion model in horses. Lesions were created in all lateral suspensory branches of 12 warmblood horses. In each horse, 2 of the 4 lesioned branches were treated daily with a multi-frequency high-power laser for 4 weeks. Color Doppler ultrasonography was performed during and after the treatment period. Six horses were euthanized 4 weeks post-surgery (short-term) and 6 were further rehabilitated until 6 months and then euthanized (long-term). High-field MRI evaluation was performed on all cadaver limbs. On ultrasound, transverse size of the lesion was significantly smaller after 2- and 3 months (*p* = 0.026 and *p* = 0.015) in the treated branches. The expected post-surgery enlargement of the lesion circumference and cross-sectional area (CSA) over time, was significantly lower in the short-term laser treated group (*p* = 0.016 and *p* = 0.010). Treated lesions showed a significantly increased Doppler signal during treatment (*p* < 0.001) compared with control. On MRI, in the short and long-term group, the CSA of the lesions was significantly smaller (*p* = 0.002), and the mean signal significantly lower in the treatment groups (*p* = 0.006). This standardized controlled study shows that multi-frequency high-power laser therapy significantly improves healing of a suspensory branch ligament lesion.

## Introduction

Tendinopathy and desmopathy are highly prevalent injuries in sports horses and are unfortunately often career-ending injuries, despite the application of numerous treatment methods. Desmitis of the suspensory ligament branches (SLB) represents ~31% of all the tendon and ligament injuries in horses, (1) requires a lengthy rehabilitation period and often leads to early ending of the athletic career (1, 2). Typically, the suspensory ligament stores and returns kinetic energy to the exercising horse and this “shock-absorbing” function explains its vulnerability to overload (3). A relatively novel treatment option in equine medicine is high-power laser therapy. In human medicine this therapy has been successfully used to address multiple orthopedic pathologies (4–7). In a single blinded randomized clinical study in human Achilles tendinopathy, high-power laser therapy significantly reduced pain (8). Both *in-vivo* and *in-vitro* studies covering a wide range of research models in different animal species have reported beneficial effects of laser treatment including increased proliferation of fibroblasts, stimulation of collagen production, increase of collagen alignment, increased tendon tensile strength, increased angiogenesis and micro- vascularization and reduction of COX-2 and pro-inflammatory mediators expression (9–11). Recently, promising results have been reported in a retrospective clinical study focusing on 150 sport horses suffering from tendon- and ligament injuries of the SDFT, DDFT, suspensory ligament and suspensory branches and treated with high-power laser therapy (12). In that study a significant improvement of lameness and ultrasound scores, beginning 2 weeks after initiation of high-power laser therapy, was observed. Moreover, re-injury rates (respectively, 16.8, 21.0, and 18.2% after 6, 12, and 24 months) was within the lower ranges of previously published studies on several other treatment modalities (13), and a rapid build-up of the rehabilitation program was possible. Re-injury rate after stem cell therapy in SDF tendinopathy was 27.4% in a study covering 113 racehorses 2 years after the onset of full exercise (14) and 18% in a study covering 168 racehorses (15). In a study covering 83 riding horses suffering from suspensory ligament desmitis treated with adipose-derived stem cells, return to full work rate was > 80% (16).

Re-injury rate of 135 horses competing in varying disciplines suffering from SDF tendinopathy varied between 42.5 and 44.4% 2 years after the onset of full exercise following conservative treatment (17).

Thus far, no standardized experimental study on the effectiveness of high-power laser therapy to treat tendon lesions in neither humans, nor horses, has been performed.

**Figure 1:**
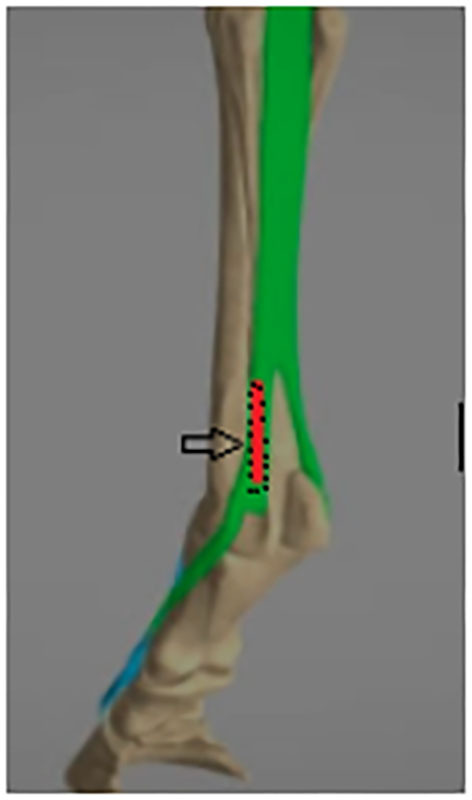
Suspensory branch mechanical lesion model anatomical view: The M. interosseus medius bufurcates into two branches: the medial and lateral suspensory branch, each of which inserts on a sesamoid bone at the palmar/plantar aspect of the fetlock. The lesion site is indicated in red.

Typically, experimental studies on equine tendon injuries include the induction of a lesion to the superficial digital flexor tendon (SDFT) (18–23), which is chosen because of the high prevalence of SDF tendinitis in racehorses (24). However, in warmblood riding horses the prevalence of suspensory ligament desmopathy is much higher compared to SDF tendinopathy (1, 12) which is related to the much different challenges posed by the typical equestrian disciplines in which warmbloods perform (25). We therefore decided to perform our experimental study in the branches of the suspensory ligament (Figure 1).

Tendon- or ligament lesions can be experimentally induced either mechanically or enzymatically by injecting substances like collagenase, in order to induce acute collagen breakdown (26, 27). Both approaches have their pros and cons, but there is no tendon lesion model available that perfectly mimics naturally occuring tendon lesions due to (chronic) overload, which is the type of lesion most often observed in horses.

Follow-up of tendon- and ligament healing in the horse can be performed in different ways. With respect to medical imaging, both ultrasound and MRI are commonly used and validated techniques to assess tendon and ligament healing in the horse (2, 18, 19).

The ultrasonographic appearance of an acute lesion is typically hypoechogenic in the acute stage as a result of inflammation, edema and disruption of collagen fibers. The proliferation phase encompasses angiogenesis and infiltration of fibroblasts resulting in an increased echogenicity. Ultrasonographically, the size of a naturally occurring acute lesion will typically increase in the first 2 weeks after injury, after which the size decreases during healing (28). In a mechanical lesion model however, as performed in the current study, the volume of the lesion is reported to further increase up to 6 weeks after lesion creation (18). Doppler ultrasonography has been validated to assess suspensory branch desmitis in the horse and allows proper visualization of neovascularization associated with tendon healing (29, 30). High field MRI is a reliable medical imaging technique to evaluate tendon and ligament integrity. On MRI, the edema in an acute lesion is characterized by a higher water content, resulting in an elevated signal on T1, T2, and Proton Density (PD) fatsat scans. During the proliferation and remodeling phases, the signal intensity decreases again (19, 31). This is the first study to apply a standardized mechanical lesion model to the suspensory branches of the horse and to evaluate the effect of high-power laser therapy in a controlled experimental lesion model in a large mammal. In follow-up of the results of the retrospective clinical study performed in the past (12), the hypothesis of the current standardized study was that high-power laser therapy improves ligament healing in the horse.

## Materials and Methods

For this study, approval of the ethical commission of the University of Ghent was obtained (LA1400077). Twelve, healthy, adult warmblood horses (age 4–12 years, 5 geldings, and 7 mares) were included. A standard orthopedic, ultrasonographic and pre-anesthetic examination was performed pre-operatively. Only healthy horses without lameness or any (sub-clinical) ultrasonographic changes on the suspensory branches were included. General appearance, soundness at the walk, appetite, body temperature, heart rate, respiratory rate as well as local clinical signs at the level of the suspensory branches such as heat, swelling and pain were recorded post-operatively on a daily basis throughout the entire study. Horses were checked for lameness, by a veterinarian and with use of an objective motion analysis system, weekly in the first 4 weeks and monthly afterwards. The horses received phenylbutazone 2.2 mg/kg PO q12h for the first 5 days post-operatively. Six horses were used for a short term evaluation and were euthanized at 4 weeks, while 6 horses were used for a long term evaluation and euthanized after 6 months.

### Surgical Procedure

**Figure 2:**
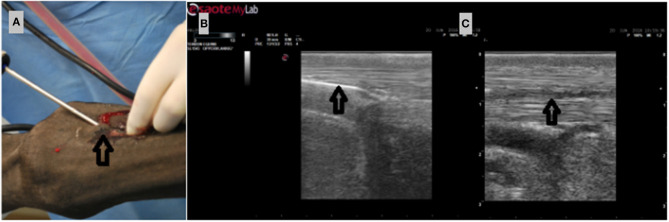
**(A)** Insertion of the shaver 1.5 cm proximal to the lateral suspensory branch attachment to the sesamoid bone. **(B)** Ultrasonographic longitudinal view of insertion of the shaver (indicated by a black arrow). **(C)** View of the induced lesion immediately after the flushing procedure (lesion canal indicated by black arrow).

Under general anesthesia, in lateral recumbency, a core lesion was created mechanically in all 4 lateral suspensory branches. The protocol was a modification of the method described by Schramme at al. (18). Briefly: a 5 mm deep incision was made 1.5 cm proximal to the distal attachment of the suspensory branch onto the sesamoid bone. Under palpatoric and ultrasonographic guidance, a 4 mm wide shaver (Arthrex Torpedo®) was inserted into the center of the lateral suspensory branch (Figure 2), and advanced proximally over a length of 4 cm. The shaver was activated (3000 rpm, oscillating, aggressive mode) and withdrawn with a twisting motion for 1 min. Afterwards, a blunt cannula was inserted (Figure 2) and the lesion was flushed with 15 ml NaCl 0.9% under ultrasonographic guidance (Figure 2). The shaving and flushing procedure was performed twice. The skin incision was then closed using a simple intra-dermal suture (Monosyn® USP 3/0). A sterile bandage was applied. Bandages were changed daily to allow inspection of the wound and application of laser therapy. From 10 days after surgery onward, no more bandages were applied.

### Laser Protocol

The device used in the current study is a high-power (class 4) multi-frequency laser (Prototype, software installed for the aim of this study, Touch Life Rehab®) with a maximal output of 15W. The laser emits light simultaneously at 4 different wavelengths between 635 and 980 nm. The laser contains a build in temperature sensor to protect the patient against skin burns. In each horse, laser therapy was applied daily on 2 of the 4 induced lesions, on clean skin, starting at day 1 after surgery for 4 consecutive weeks. In each horse, the treatment location was randomly allocated either to the right front and left hind limb, or to the left front and right hind limb, equally divided across the study group. Treatment was performed moving the laser handpiece in a linear fashion upon the lesion area with the handpiece held perpendicular to the skin at a distance of 0.5 cm. Duration of this laser protocol was 20 min.

### Exercise Protocol

The exercise protocol is summarized in Table 1. Starting at day 1 post-operatively, the horses were lunged at the trot for 1 week, altering direction every 5 min. This was performed daily or until an obvious lameness was present at the trot, whichever occurred first. After the first week, the horses were hand-walked on a hard surface for 20 min on a daily basis until week 4 for the short term group and month 3 for the long term group. For the long term group, trotting exercise with gradually increasing intensity on a soft surface was started 3 months after surgery.

**Table 1:** Exercise protocol.

	**Walk (mins)**	**Trot (mins)**	**Canter (mins)**
D 1	20	10	–
D 2–4	20	20	–
D 5–7	20	30	–
W 2–M 3	20	–	–
M 3–M 5	20	Increasing by 2 min weekly	–
M 5–M 6	20	20	Increasing by 2 min weekly

### Ultrasound Examination

**Figure 3:**
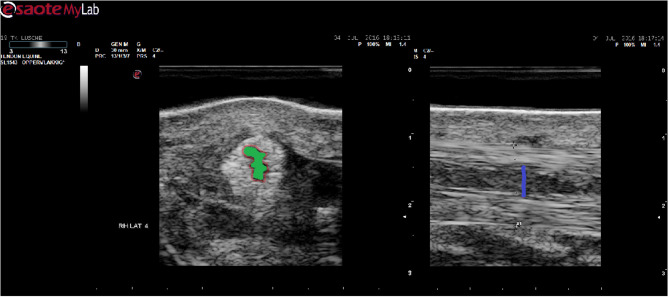
Schematic overview of ultrasonographic measures: The circumference of the lesion indicated by a red line (left panel) and the CSA by green. Transverse size on a longitudinal view indicated by a blue line (right panel).

Ultrasound and color Doppler imaging (with a 10 Herz linear probe, Esaote Mylab alfa®) were started with a baseline measurement before surgery (D0) Follow-up measurements started on day 1 after surgery (D1) and were consistently performed by the same operator (MP). During the laser treatment period of 4 weeks, ultrasonography was performed on a weekly basis (W1–W4). In the long term follow-up group, examinations were continued on a monthly basis until the end of the study (M2–M6). During the ultrasonographic examination, images, and video clips were recorded using a standardized protocol. In the standing limb, video clips of the lateral suspensory branch were made in a transverse and longitudinal view from the bifurcation of the suspensory ligament down to the distal attachment of the branch onto the sesamoid bone. Still images were made at 2, 4, and 6 cm proximal to the distal attachment of the branch onto the sesamoid bone. These images and video clips were recorded by the same operator (MP) and later evaluated by a blinded board-certified veterinary radiologist (KV). The following parameters were assessed: Cross-sectional area (CSA), circumference, and transverse size of the lesion and ligament, peri-ligamentous swelling, echogenicity and color Doppler signal intensity. The transverse size of the lesion was measured on a longitudinal view (one measurement per lesion, Figure 3), whereas the circumference and CSA of the lesion and tendon was measured on transverse scans (measurements at 3 different heights, 2, 4, and 6 cm, Figure 3). Echogenicity was calculated as a percentage of the pixel intensity of the lesion compared to the pixel intensity of the non-injured portion of the suspensory branch Pixels were count with aid of Osirix® software. On a transverse ultrasonographic picture at 4 cm height a region of interest (ROI) was drawn in the lesion and in the non-injured part of the ligament on the same image. A percentage was calculated by dividing the amount of pixels present in the ROI of the lesion by the amount of pixels present in the ROI of the non-injured site of the ligament. The color Doppler signal was recorded on a flexed limb at 4 cm height, and was scored in 6 grades, where 0 stands for an absent signal, and 5 for a strongly increased signal. To make the outcome variable binary, a cut-off value of a grade of > 1 was used to define an increased signal.

### MRI Examination

A high field MRI examination was performed on the cadaver limbs after euthanasia, using a 3 Tesla MRI (Achieva Philips®). A dedicated 8 channel human knee coil was used for positioning. In a preliminary study on a series of cadaver limbs, the optimal positioning of the limbs was defined, in order to minimize occurrence of the magic angle effect. The magic angle artifact (related to spectral fat suppression, short echo time and/or fractional anisotropy of the tendon fibers due to positioning) was not observed in T1 Fat Suppressed (FS) axial images (TSE, 2 mm) and Proton Density (PD) FS axial images (TSE, 2 mm) with the limbs positioned with the dorsal side of the limb turned toward the table, and the distal side of the limb inserted first into the MRI machine. The morphological scoring, on the T1 scans, was performed by 3 independent blinded observers on a post-processing-console (Extended Brilliance Workspace Philips®). CSA and circumference of the lesion and of the ligament were measured at 3 different levels: 2, 4, and 6 cm proximal to the sesamoid bone. The quantitative mean signal was measured on PD fatsat scans with use of a MR-console (Syngo HR Acquisition Workplace Siemens®). All 3 observers repeated the measurements 3 times.

### Statistical Analysis

For the ultrasonographic measurements of the transverse size of the lesion and the transverse size of the ligament, an independent *t-*test was performed on each of the occasions (D1–M6) independently to compare treatment with control group. The difference of CSA and circumference of the lesion between the treatment and control group was assessed over all time points and all of the 3 measurement heights by means of a multivariate linear mixed model. The difference of echogenicity between the treatment and control group was assessed over all time points by means of a multivariate linear mixed model and a *post-hoc* Sheffe test. The evolution of the transverse size, CSA and circumference of the lesion between week 1 and week 4 (W1–W4) and week 1 and month 6 (W1–M6) was analyzed by means of a multivariate linear mixed model correcting for horse and limb. The difference in binary Doppler signal between treatment and control group over all time periods was analyzed by means of logistic regression. The difference in CSA and mean signal of the lesion in MRI between treatment and control group, as evaluated at week 4 and month 6, was analyzed by means of a multivariate linear mixed model accounting for the measurement height, horse, limb, and the observer effect.

## Results

### Lesion Model and Post-operative Follow-Up

No intra- or post-operative complications were observed in any of the 12 horses. There was no swelling, heat or pain at palpation on the first day after surgery (D1). Mild to moderate swelling, heat and pain at palpation was present in all horses after 1 week of lungeing exercise (W1). All horses were sound at walk throughout the entire study. Moderate lameness on one limb at a trot occurred in 3/12 horses during exercise in the first week after surgery. One horse developed a pulmonary infection during the first week of treatment and received antibiotics for 1 week (Trimetoprim 120 mg/Sulfadiazin 600 mg, Synutrim®, 30 mg/kg). Two horses developed a small seroma underneath the surgical incision. Disinfecting bandages with iodine were applied and healing occurred uneventfully. The exercise protocols did not need to be altered. There were no skin irritations nor other complications due to laser therapy and all horses tolerated the therapy well.

### Ultrasound and Color Doppler

**Figure 4:**
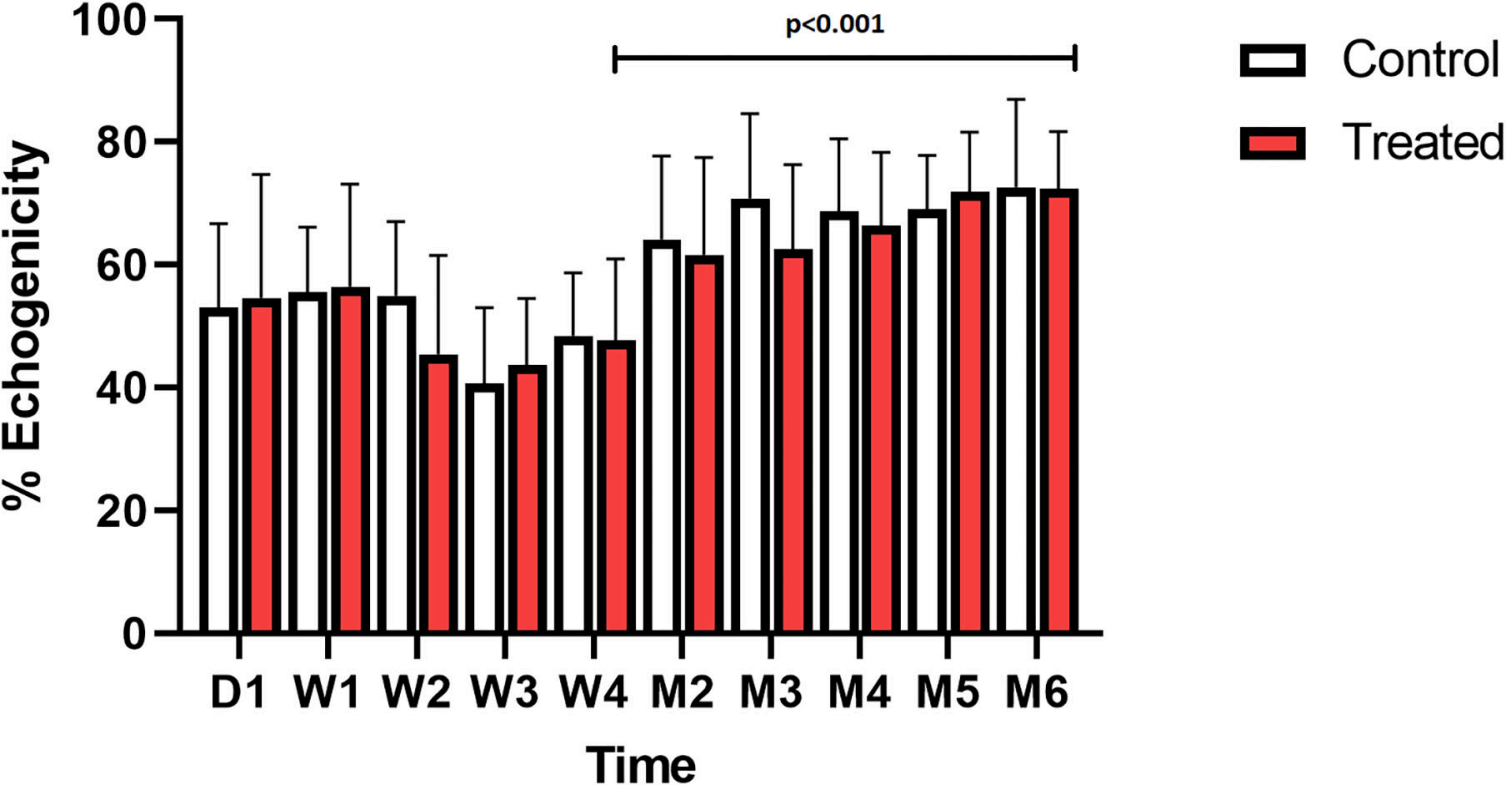
Ultrasound: mean percentage with SD of echogenicity of the lesion, compared to the non-injury site of the suspensory branch, over time; treated (red) vs. control (white).

**Figure 5:**
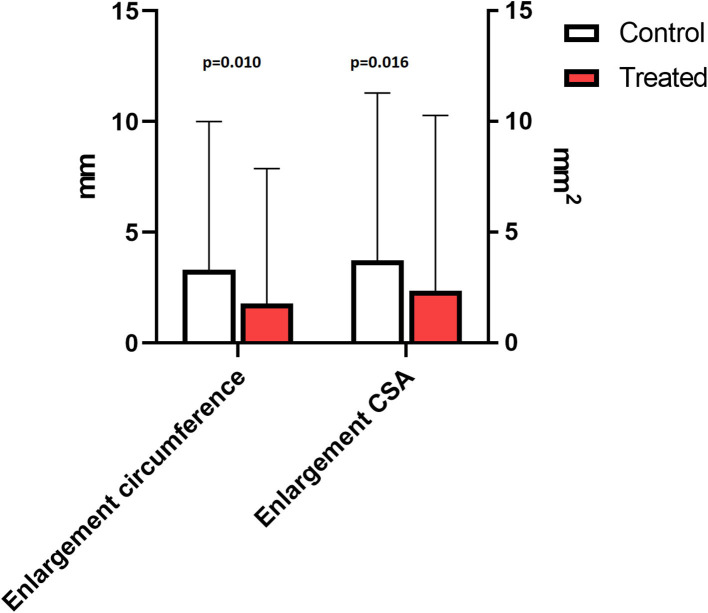
Ultrasound: mean enlargement with SD of the lesion of all measured heights until end of the treatment period in the short-term study (W4 compared to W1): circumference (mm)- and CSA (mm^2^) of the lesion; treated (red) vs. control (white).

**Figure 6:**
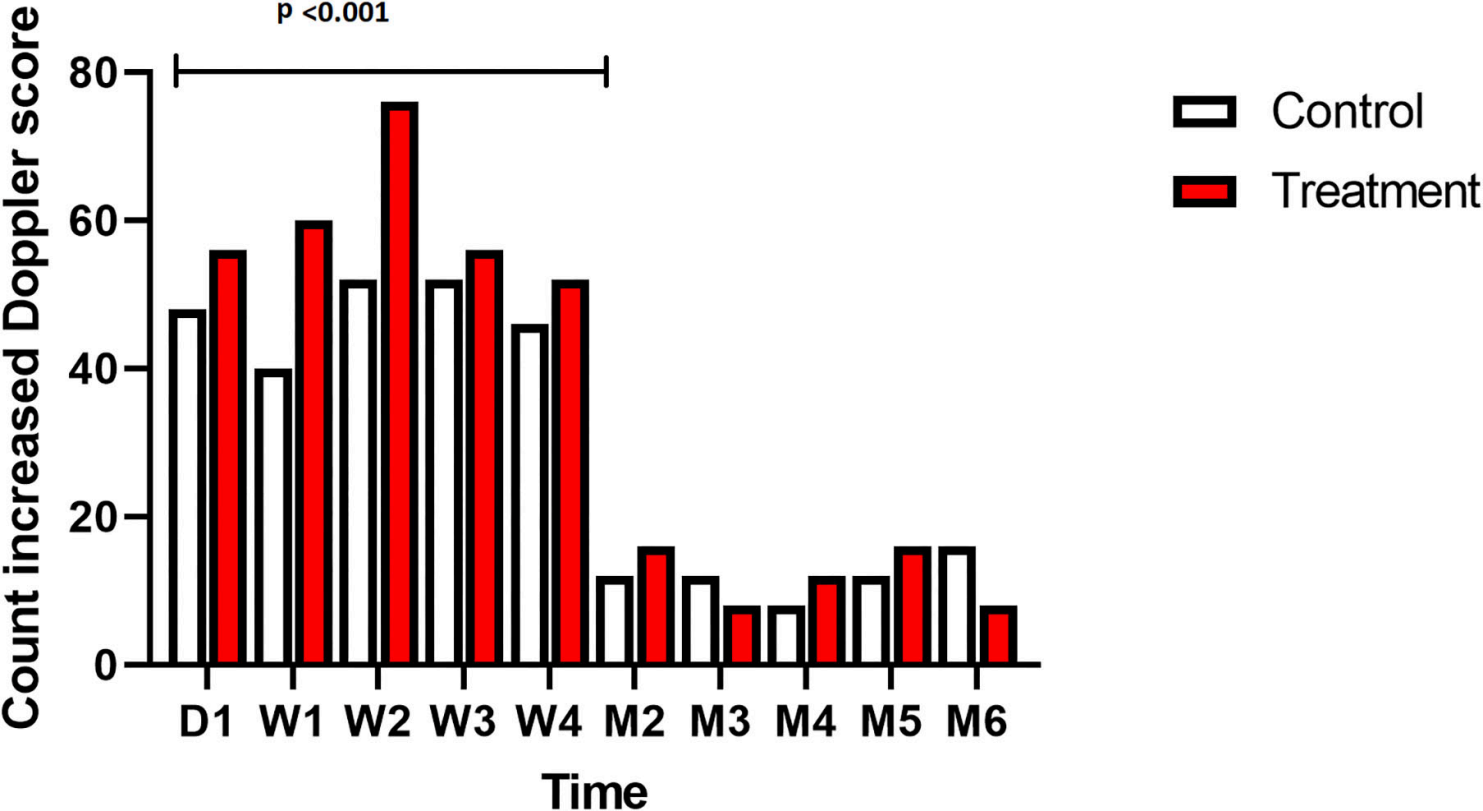
Ultrasound: total count of increased Doppler scores in all horses and all limbs treated (red) vs. control (white) over time.

On D1, the first day after surgery, lesions were very small in size. For both control and treatment groups the lesion increased progressively in circumference, transverse size and CSA for 3–4 months after lesion creation, and started to decrease in size thereafter (Table 2). The echogenicity of all the lesions initially decreased until W3, followed by a significant increase after W4, compared to W3 (*p* < 0.001, Figure 4, Supplementary File 1). The transverse size of the lesion, measured on a longitudinal view was smaller in the laser treated group, this difference was significant at M2 (3.5 vs. 4.3 mm, *p* = 0.026) and M3 (4.2 vs. 4.6 mm, *p* = 0.015). The enlargement over time (W4 compared to W1) of both the circumference and CSA of the lesion in the short-term group was significantly smaller in the laser treated limbs compared to control limbs (*p* = 0.010 and *p* = 0.016, Figure 5). There was a significantly increased color Doppler signal in the laser treated suspensory branch during the treatment period (until W 4) compared to the non-treated control branches (*p* < 0.001, Figure 6).

**Table 2:** Size of the lesion on ultrasound over time (mean and SD, significant differences between groups marked with *) Averages for height 2, 4, and 6 cm are used for CSA and circumference.

**T**	***n***	**Average CSA (mm^**2**^) control**	**Average CSA (mm^**2**^) treatment**	**Average circumference (mm) control**	**Average circumference (mm) treatment**	**Transverse size (mm) control**	**Transverse size (mm) treatment**
D1	12	3.1 SD 5.1	3.3 SD 5.6	5.0 SD 5.3	4.9 SD 5.5	3.2 SD 1.5	3.3 SD 1.8
W1	12	3.9 SD 4.9	5.3 SD 6.2	6.0 SD 5.9	7.0 SD 7.1	2.9 SD 0.9	3.5 SD 1.5
W2	12	5.1 SD 6.9	5.1 SD 5.8	7.6 SD 7.9	7.5 SD 7.5	3.2 SD 1.5	3.1 SD 1.3
W3	12	4.9 SD 5.9	4.8 SD 6.0	7.3 SD 7.1	6.9 SD 7.5	3.4 SD 1.6	3.0 SD 1.6
W4	12	6.3 SD 7.4	5.9 SD 6.7	8.9 SD 8.9	7.8 SD 7.7	3.4 SD 1.3	3.5 SD 1.1
M2	6	7.5 SD 9.3	7.2 SD 8.8	8.3 SD 8.9	7.2 SD 8.1	4.3* SD 1.9	3.5* SD 0.9
M3	6	18 SD 17.9	10.9 SD 11.3	13.1 SD 12.4	10.6 SD 9.6	4.6* SD 1.9	4.2* SD 1.0
M4	6	12.7 SD 12.3	11.1 SD 12.2	12.0 SD 10.7	11.1 SD 12.2	4.5 SD 1.6	4.4 SD 1.9
M5	6	12.7 SD 14.9	10.9 SD 13.1	12.7 SD 14.9	10.9 SD 13.1	5.9 SD 3.0	3.7 SD 1.9
M6	6	11.7 SD 12.9	9.4 SD 10.3	9.6 SD 9.8	9.4 SD 10.3	3.9 SD 2.2	3.6 SD 1.3

### MRI

**Figure 7:**
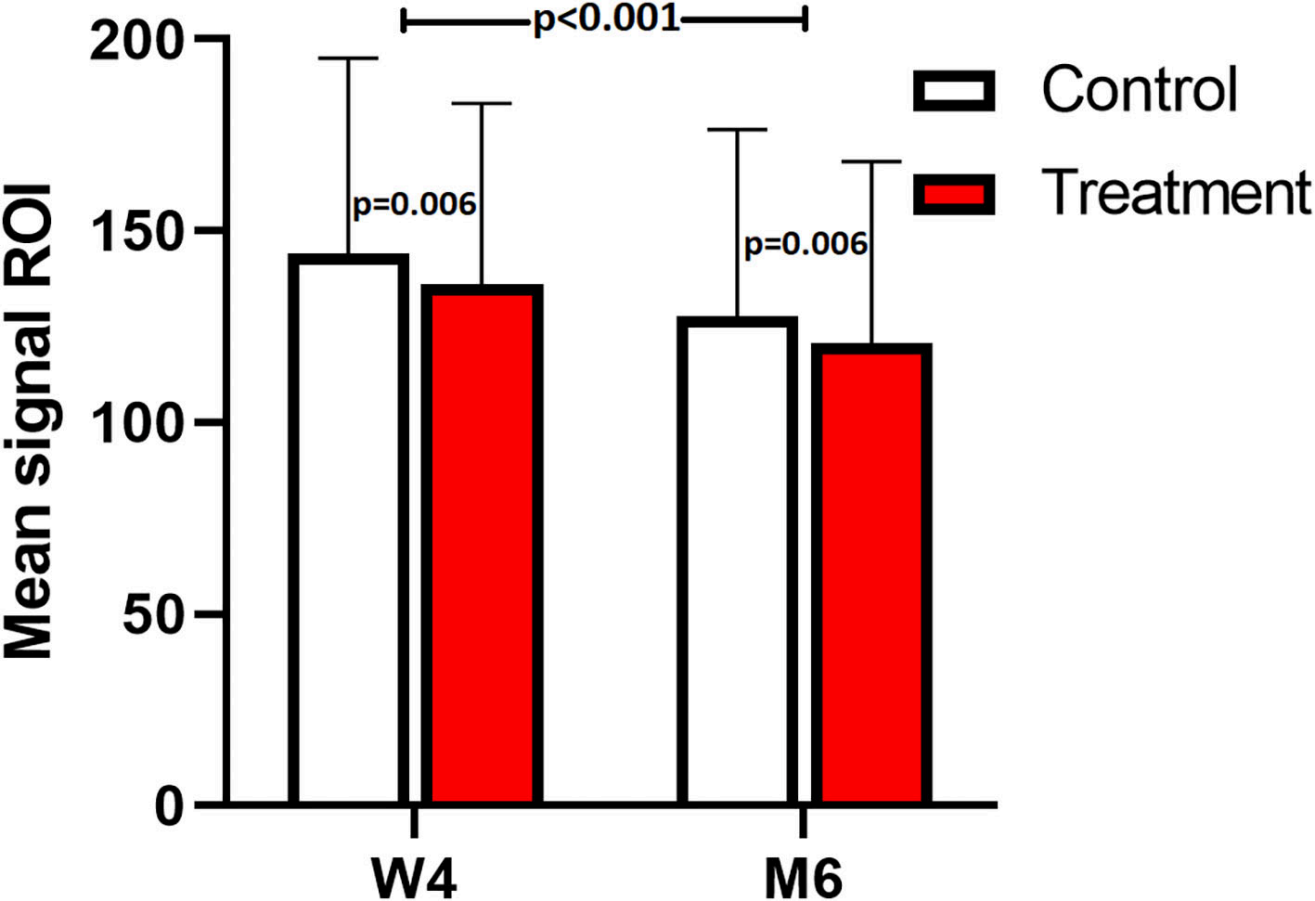
MRI: mean signal with SD of all measured heights of the short-term (W4) and long-term (M6) studies; treated (red) vs. control (white).

**Figure 8:**
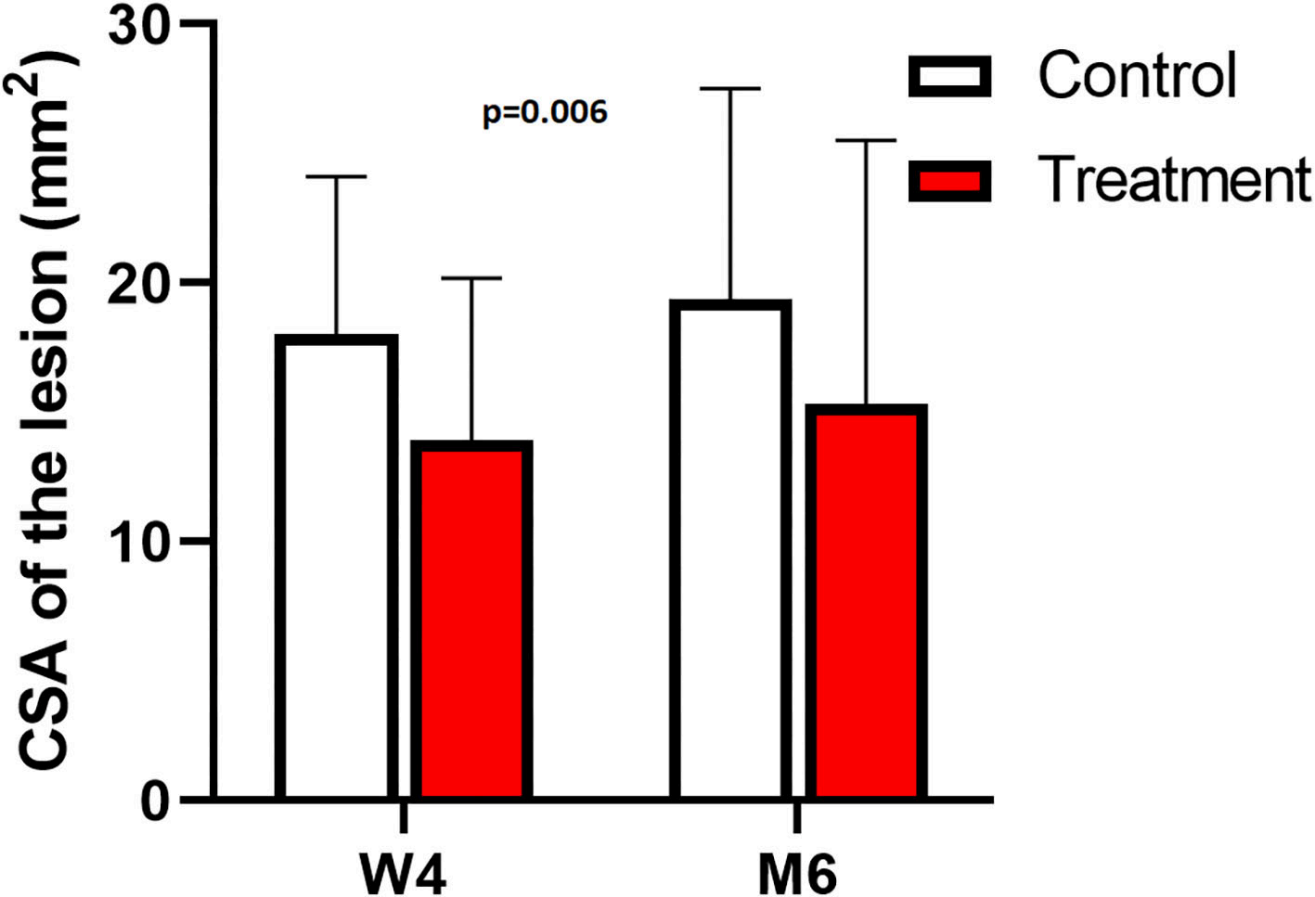
MRI: mean CSA with SD of the lesion at height 4 cm (in mm^2^) for the short-term (W4) and long-term (M6) studies; treated (red) vs. control (white).

There were no significant differences between measurements of the 3 different observers (*p* = 0.77) and there was a significant difference between mean signal on a PD fatsat scan in the short-term group vs. the long-term group, with a lower signal in the long-term group, for both treatment and control limbs (ROI 124.2 vs. 140.1, *p* < 0.001, Figure 7, Supplementary File 2). The signal intensity was significantly lower at height 6 vs. 2 and 4 cm in both treatment and control group (*p* < 0.001). The mean signal was significantly lower in the treated suspensory branches, in both short and long-term group, vs. the contra-lateral untreated branch (*p* = 0.006, Figure 7). The CSA of the lesion on a T1 scan at height 4 cm was significantly smaller in the treated limbs, in both short- and long term group, vs. control limbs (14.6 vs. 18.7 mm^2^, *p* = 0.002, Figure 8).

## Discussion

High-power laser therapy in horses has thus far only been studied in a clinical setting by means of a retrospective study (12). In human subjects, also only clinical retrospective studies are available (4–6, 8). In horses, short-term outcome in terms of improvement of lameness and ultrasonographic appearance and long-term outcome in terms of re-injury rate have shown promising results (12). This is the first standardized experimental study to assess the effect of high-power laser therapy on ligament healing in horses. From a comparative physiological point of view, the composition and bio-mechanical function of the distal suspensory branches in horses show great similarity with the Achilles tendon in humans (32, 33). Therefore, the results of the current study can, at least to some degree, be extrapolated to the human Achilles tendon, keeping in mind that both load and strain differ between horse and human. Our results indicate that standardized lesion induction in the suspensory branch of the horse could be performed without complications with no swelling, heat or pain at palpation detectable during the clinical examination on the first day after surgery. Such signs are typically manifested directly after occurrence of clinical acute desmitis in the horse (1–3, 24, 28). A possible explanation for absence of these symptoms might be a more modest inflammatory reaction in these mechanically induced lesions compared to the clinically often observed acute presence of (chronic) overload lesions. The inflammatory response was therefore stimulated by exercising the horses, starting at day 1 after surgery, as described by Schramme et al. (18). Mild to moderate swelling, heat, and pain at palpation were present in all horses after 1 week of lunging exercise. Similarly, Bosch et al. (20) described the necessity to combine of enzymatic and mechanical stimulation to propagate inflammation and enlargement of experimentally induced tendon lesions. In the current study, it was chosen to apply laser treatment starting the day after lesion induction because this matches with how this treatment technique is applied in clinical practice. Future standardized studies could look into differential effects of different treatment protocols of laser therapy on ligament healing.

The medical imaging techniques that were applied in the current study to monitor tendon lesions provided parameters on signs of ligament healing. Ultrasound is typically used in equine veterinary practice to follow-up tendon- and ligament lesion healing. Three different commonly used measuring techniques were applied: (1) the transverse size of the lesion on a longitudinal view, (2) the circumference of the lesion on a transverse view, and (3) the CSA on a transverse view.

**Figure 9:**
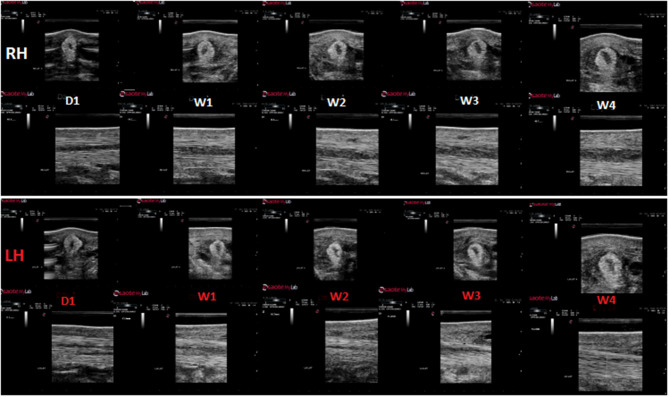
Example of Ultrasonographic evolution of the lesion in the control limb (upper panel) vs. laser treated limb (lower panel) in the same horse.

This is the first standardized controlled study to evaluate the effects of high-power laser treatment on ligament healing in horses. The transverse size of the lesion was significantly smaller in the laser treated group at 2 and 3 months. While the lesions generally developed slightly differently in each limb in the first week (exercise week), we also evaluated the enlargement of the lesion of each limb after the initial exercise week (W1) and until the end of the study (W4 in the short-term and M6 in the long term follow-up group). An initial phase of lesion enlargement was followed by a phase of lesion size reduction (Figure 9). This has also been reported in clinical non-experimental tendon lesions (25), as well as in other experimental tendon lesion studies. Other studies using a mechanical tendon lesion model in the SDFT of the horse have reported an initial tendon lesion enlargement (22) of up to 6 weeks duration (18), followed by a progressive lesion shrinking phase (18). In our study echogenicity of the lesion decreased in the first 3 weeks, followed by a significant increase of echogenicity after W4 in both control- and treatment group. This means that the proliferation phase, which encompasses angiogenesis and infiltration of fibroblasts resulting in an increased echogenicity, occurred before lesions started to reduce in size. Transverse size, circumference and CSA of the lesion increased until, respectively, M3 and M4 (long-term study group). Analysis of the enlargement of the lesion in the short-term group showed a significantly less pronounced increase in circumference (*p* = 0.010) and CSA (*p* = 0.016) from week 1 until week 4 for the laser treated lesions compared to the control lesions. Reduction of lesion propagation is an outcome parameter evaluating effectiveness of specific treatment modalities on tendon healing. In a study using a standardized lesion model 10 days of cast immobilization was shown to effectively reduce lesion propagation compared to bandaging (22). The evolution of the size of the lesions on ultrasound in the current study agreed with an earlier study using a mechanical tendon lesion model in the SDFT (20) and showed more rapid and pronounced tendon healing in the laser treated lesions. Whether this also resulted in increased tensile strength cannot be concluded from the current experiment. An indirect indication may be that in the previously reported clinical study in 150 sport horses, re-injury rate was within the lower ranges of previously published studies on several other treatment modalities, and a rapid rehabilitation was possible in laser treated horses (12). The Doppler signal was significantly increased (grade 2–5) in the laser treated group during the treatment period. Doppler ultrasonography is being used as add-on to plain ultrasound to assess pathophysiological processes seen in the suspensory branch of the horse (29). An increased Doppler signal is seen during the acute and proliferative phase of tendinitis (29, 34) and is considered a sign for active tendon healing. Increased angiogenesis and manifestation of increased collateral micro-circulation have been reported after laser therapy in experimental rodent studies (11).

In the earlier mentioned clinical study evaluating high-power laser therapy in 150 sport horses (12), significant improvement of ultrasonographic appearance was seen directly after 2 weeks of laser therapy, and 4 weeks later. However, the improvement was more pronounced directly after laser therapy than at the later point in time. In line herewith, ultrasonographic parameters such as enlargement of the lesion and Doppler signal significantly differ between treatment and control group in the short-term group. These differences were present both during and after laser therapy, but were more pronounced during the treatment period in the current study, as has been described for collagenase-induced Achilles tendinitis in rats (9). Ultrasonographic appearance is unable to predict quality of the healing tendon tissue, but histopathological follow-up would provide data at the ultra-structural level. Bosch et al. (21) evaluated the effect of Platelet Rich Plasma (PRP) injection on tendon healing at ultra-structural level, using a standardized mechanical lesion model in the SDFT of the horse. They demonstrated repair tissue in the PRP treatment group with superior organization of the collagen network, increased metabolic activity, and higher strength at failure in bio-mechanical testing compared to controls (21). To support findings of diagnostic imaging further histological- and biomechanical evaluations of the suspensory branch samples are warranted to assess the structural and functional properties of the repair tissue.

**Figure 10:**
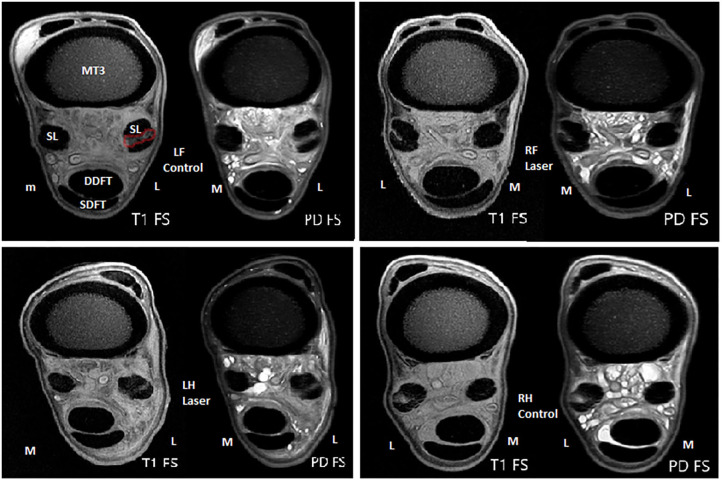
Example of MRI evaluation at height 4 cm (T1- and PD Fatsat scans) of the control- and treated limbs at W4 (short term study) of the same horse. Metacarpus 3 (MC3), deep digital flexor tendon (DDFT), superficial flexor tendon (SDFT), and suspensory branches (SL), and the lesion (red) are marked in the first image.

Our MRI results support the findings of the ultrasound and Doppler evaluations. The mean signal intensity on MRI (PD fatsat) was significantly lower in the laser treated group compared with the non-treated control limbs in both the short and long term-study (*p* = 0.006). A tendon or ligament lesion is characterized by an increased mean signal on a PD fatsat scan (Figure 10), and in the proliferation and remodeling phases, the signal decreases again (31). Since the mean signal was significantly lower in the laser treated branches in both the short- and long-term study, this is suggesting better tendon healing in the laser treated group.

The measured CSA of the lesion on MRI, both the short and long-term, was significantly smaller in the treated group, however only at a height of 4 cm (*p* = 0.002). This can possibly be explained by specific features of the applied lesion model. The mechanically created lesion was originally 4 cm in length. The shaver was inserted 2 cm proximal to the sesamoid bone and subsequently advanced up to 6 cm. This entails that “height 4 cm” actually represents “the middle” of the lesion. Apparently, it is at that level that the difference in ligament healing on MRI between groups is most obvious. In both the treatment and control groups, the mean signal was significantly lower in the long-term group (chronic stage) vs. short-term group (acute stage), while the CSA of the lesion was not smaller in the chronic stage compared to the acute stage. This phenomenon has also been described in a SDFT lesion model, where the signal decreased with the age of the lesion (19). To assess healing stage on MRI, the mean signal intensity on a PD fatsat scan seems a more useful measure than the CSA of the lesion on a T1 scan. Limitation in this study is the lack of a baseline MRI, for ethical reasons, to evaluate evolution of each lesion throughout the study, as is performed with ultrasonographic examination. While a high field MRI could only be performed under general anesthesia, the benefit of such a baseline MRI did not outweigh the extra invasion for the experimental animals.

The results of this study indicate a positive effect of high-power laser therapy on tendon healing in horses based on ultrasound, Doppler and high field MRI follow-up. To evaluate the effect of high-power laser therapy on both the ultra-structural and functional level, further histopathological and bio-mechanical follow-up of high-power laser treated and untreated lesions is required. Given the very similar composition and bio-mechanical function of the distal suspensory branches in horses and the Achilles tendon in humans, results of this study might be extrapolated to support the use of high-power laser therapy in Achilles tendon lesions, often encountered in human athletes (8, 33), although more species-specific research on that particular lesion is needed.

## Conclusion

In this standardized study, it was shown that multi-frequency high-power laser therapy is effective for enhancing healing of the suspensory branch ligament in the horse. On ultrasound, laser treated lesions showed significant better results: transverse size at M2 and M3 was smaller, enlargement of the lesions in the short-term group was lower and an increased Doppler signal during treatment period was present in the laser treated group compared to control. On MRI, in the short- and long term group, the lesions were significantly smaller, and the mean signal significantly lower in the treatment groups. Further histological- and biomechanical evaluations of the suspensory branch samples are warranted to assess the structural and functional properties of the repair tissue.

## Data Availability Statement

The datasets generated for this study are available on request to the corresponding author.

## Ethics Statement

The animal study was reviewed and approved by Ethical Commission of the University of Ghent.

## Author Contributions

We confirm that the manuscript has been read and approved by all named authors and that there are no other persons who satisfied the criteria for authorship but are not listed. MP, AM, KV, RW, MHWK, AL, and CD contributed conception and design of the study. MP, AM, MO, BV, and LP produced and or/collected the data. MP, KV, MK, BV, JD, and CD interpreted the data. MP, AM, KV, RW, MO, MK, BV, JD, MHWK, AL, LP, and CD wrote and/or revised the manuscript. All authors contributed to the article and approved the submitted version.

## Conflict of Interest

The authors declare that the research was conducted in the absence of any commercial or financial relationships that could be construed as a potential conflict of interest.
