# Supplementary material for: High-Power Laser Therapy Improves Healing of the Equine Suspensory Branch in a Standardized Lesion Model
Source: Front Vet Sci. 2020 Sep 3;7:600. doi: 10.3389/fvets.2020.00600 (PMC7494822; doi:10.3389/fvets.2020.00600)
Supplement: Supplementary file 1 [file Table_1.DOC]

Supplementary file 1: Ultrasonographic measurements over time

| T | N |  | Mean CSA tendon (mm^2^) ±SD | Mean transverse size tendon (mm) ±SD | Mean peritendinous sweling (mm) ±SD |
| --- | --- | --- | --- | --- | --- |
| D1 | 12 | Control | 88.2  ±24.1 | 10.3  ±0.2 | 2.2  ±1.1 |
|  |  | Treatment | 90.7  ±23.0 | 97.0  ±0.2 | 2.2  ±1.2 |
| W1 | 12 | Control | 102.6  ±28.3 | 11.1  ±0.2 | 2.9  ±1.2 |
|  |  | Treatment | 104.2  ±29.2 | 11.3  ±0.2 | 3.3  ±2.1 |
| W2 | 12 | Control | 108,0  ±26.8 | 11.3  ±0.1 | 3.3  ±1.7 |
|  |  | Treatment | 109.6  ±31.3 | 11.4  ±0.2 | 4.1  ±2.6 |
| W3 | 12 | control | 108.0  ±27.3 | 11.6  ±0.1 | 3.3  ±1.8 |
|  |  | treatment | 110.3  ±28.1 | 11.4  ±0.2 | 3.6  ±2.0 |
| W4 | 12 | control | 108.3  ±24.1 | 11.7  ±0.1 | 3.3  ±2.0 |
|  |  | treatment | 110.0  ±27,28 | 11.6  ±0.2 | 3.6  ±2.1 |
| M2 | 6 | control | 105.5  ±28.8 | 12.0  ±0.1 | 3.3  ±1.5 |
|  |  | treatment | 106.0  ±28.9 | 11.5  ±0.1 | 3.4  ±1.4 |
| M3 | 6 | control | 103.9  ±25.9 | 11.4  ±0.2 | 3.1  ±1.7 |
|  |  | treatment | 99.3  ±26.8 | 11.5  ±0.1 | 2.9  ±1.1 |
| M4 | 6 | control | 101.5  ±26.6 | 12.6  ±0.3 | 3.0  ±1.7 |
|  |  | treatment | 99.8  ±24.9 | 11.5  ±0.1 | 2.7  ±1.1 |
| M5 | 6 | control | 99.5  ±27.2 | 10.9  ±0.1 | 2.4  ±1.2 |
|  |  | treatment | 100.6  ±29.8 | 11.1  ±0.1 | 2.6  ±1.2 |
| M6 | 6 | control | 97.2  ±30.4 | 10.6  ±0.1 | 3.3  ±2.0 |
|  |  | treatment | 100.8  ±32.2 | 10.7  ±0.4 | 2.8  ±1.4 |
