# Supplementary material for: High-Power Laser Therapy Improves Healing of the Equine Suspensory Branch in a Standardized Lesion Model
Source: Front Vet Sci. 2020 Sep 3;7:600. doi: 10.3389/fvets.2020.00600 (PMC7494822; doi:10.3389/fvets.2020.00600)
Supplement: Supplementary file 2 [file Table_2.DOC]

Supplementary file 2: MRI measurements short term and long term study

| Study | Measure height | Group | Mean signal (ROI) ± SD | Mean CSA lesion (mm^2^) ± SD | Mean Circumference lesion (mm) ± SD | Mean CSA tendon (mm^2^) ± SD |
| --- | --- | --- | --- | --- | --- | --- |
| Short term  W4 (n=6) | 2cm | control | 121.5  ±56.0 | 11.0  ±5.7 | 25.6  ±12.8 | 135.2  ±21.1 |
|  |  | treatment | 120.4  ±0.0 | 12.1  ±5.9 | 27.0  ±10.4 | 140.5  ±27.5 |
|  | 4cm | control | 164.6  ±54.5 | 18.0  ±6.1 | 28.8  ±9.6 | 111.5  ±18.1 |
|  |  | treatment | 150.9  ±46.8 | 13.9  ±6.3 | 26.4  ±9.5 | 111.3  ±24.2 |
|  | 6cm | control | 145.9  ±38.1 | 15.0  ±9.9 | 26.7  ±15.5 | 105.3  ±19.3 |
|  |  | treatment | 136.8  ±38.5 | 10.9  ±5.7 | 23.6  ±8.9 | 103.9  ±22.5 |
| Long term  M6 (n=6) | 2cm | control | 149.4  ±54.7 | 10.1  ±12.7 | 17.4  ±26.2 | 130.5  ±21.5 |
|  |  | treatment | 145.8  ±45.8 | 14.5  ±10.6 | 26.0  ±23.7 | 128.1  ±18.2 |
|  | 4cm | control | 126.8  ±42.2 | 19.4  ±8.1 | 30.6  ±18.0 | 108.3  ±16.0 |
|  |  | treatment | 121.6  ±47.8 | 15.3  ±10.2 | 24.1  ±18.3 | 104.7  ±12.3 |
|  | 6cm | control | 106.7  ±38.3 | 7.8  ±7.5 | 15.3  ±16.5 | 95.0  ±14.6 |
|  |  | treatment | 95.2  ±34.1 | 5.8  ±7.3 | 11.9  ±17.2 | 93.7  ±15.2 |
